# Amniotic Fluid Glucose Concentration: A Marker for Infection in Preterm Labor and Preterm Premature Rupture of Membranes

**DOI:** 10.1155/S1064744994000025

**Published:** 1994

**Authors:** Gary A. Dildy, Mark D. Pearlman, Leon G. Smith, Guillermo Tortolero-Luna, Sebastian Faro, David B. Cotton

**Affiliations:** 1 Divisions of Maternal-Fetal Medicine and Infectious Diseases Department of Obstetrics and Gynecology Baylor College of Medicine Houston TX USA; 2 Perinatal Center Utah Valley Regional Medical Center 1034 North 500 West Provo UT 84604 USA

## Abstract

Amniotic fluid Gram stain and culture have been utilized as laboratory tests of microbial invasion of the amniotic cavity. The Gram stain of amniotic fluid has a low sensitivity in the detection of clinical infection or microbial invasion of the amniotic cavity, and amniotic fluid culture results are not immediately available for management decisions. Glucose concentration is used to diagnose infection in other sites such as cerebrospinal fluid.

*Objective:* The purpose of this study was to evaluate the usefulness of amniotic fluid glucose concentration in detecting microbial invasion of the amniotic cavity associated with preterm labor and preterm premature rupture of membranes.

*Methods:* Amniocentesis was performed in 60 women with preterm labor and/or preterm premature rupture of membranes. Gram stain and culture for *Mycoplasma hominis*, *Ureaplasma urealyticum*, aerobic, and anaerobic bacteria were performed. Subjects were studied prospectively for the development of positive amniotic fluid cultures and the development of clinical chorioamnionitis.

*Results:* The diagnosis of clinical chorioamnionitis was made in 25% (15/60) of women entered into the study. Low amniotic fluid glucose concentration Was considered < 15 mg/dl. The sensitivity, specificity, and positive predictive value of low amniotic, fluid glucose concentration to predict clinical chorioamnionitis were 73.3%, 88.1%, and 68.8% respectively, while positive amniotic fluid culture, hada sensitivity of 43.8%, specificity of 79.5%, and positive predictive value of 43.8%.

*Conclusions:* Amniotic fluid glucose concentration was more sensitive in predicting chorioamnionitis than either Gram stain or culture. Amniotic fluid glucose concentration was better in predicting clinical chorioamnionitis than predicting positive amniotic fluid culture results. Gestational age-dependent normal ranges and pathologic conditions that may alter amniotic fluid glucose concentrations should be considered when interpreting amniotic fluid glucose values to diagnose microbial invasion of the amniotic cavity.


Infectious Diseases in Obstetrics and Gynecology 1:166–172 (1994)

© 1994 Wiley-Liss, Inc.

### Amniotic Fluid Glucose Concentration: A Marker for Infection in Preterm Labor and Preterm Premature Rupture of Membranes

Gary A. Dildy, Mark D. Pearlman, Leon G. Smith, Guillermo Tortolero-Luna, Sebastian Faro, and David B. Cotton

*Divisions of Maternal-Fetal Medicine and Infectious Diseases, Department of Obstetrics and Gynecology, Baylor College of Medicine, Houston, TX*

### Abstract

Amniotic fluid Gram stain and culture have been utilized as laboratory tests of microbial invasion of the amniotic cavity. The Gram stain of amniotic fluid has a low sensitivity in the detection of clinical infection or microbial invasion of the amniotic cavity, and amniotic fluid culture results are not immediately available for management decisions. Glucose concentration is used to diagnose infection in other sites such as cerebrospinal fluid.

*Objective:* The purpose of this study was to evaluate the usefulness of amniotic fluid glucose concentration in detecting microbial invasion of the amniotic cavity associated with preterm labor and preterm premature rupture of membranes.

*Methods:* Amniocentesis was performed in 60 women with preterm labor and/or preterm premature rupture of membranes. Gram stain and culture for *Mycoplasma hominis, Ureaplasma urealyticum,* aerobic, and anaerobic bacteria were performed. Subjects were studied prospectively for the development of positive amniotic fluid cultures and the development of clinical chorioamnionitis.

*Results:* The diagnosis of clinical chorioamnionitis was made in 25% (15/60) of women entered into the study. Low amniotic fluid glucose concentration was considered < 15 mg/dl. The sensitivity, specificity, and positive predictive value of low amniotic fluid glucose concentration to predict clinical chorioamnionitis were 73.3%, 88.1%, and 68.8% respectively, while positive amniotic fluid culture, had a sensitivity of 43.8%, specificity of 79.5%, and positive predictive value of 43.8%.

*Conclusions:* Amniotic fluid glucose concentration was more sensitive in predicting chorioamnionitis than either Gram stain or culture. Amniotic fluid glucose concentration was better in predicting clinical chorioamnionitis than predicting positive amniotic fluid culture results. Gestational age-dependent normal ranges and pathologic conditions that may alter amniotic fluid glucose concentrations should be considered when interpreting amniotic fluid glucose values to diagnose microbial invasion of the amniotic cavity. © 1994 Wiley-Liss, Inc.

Key words

Chorioamnionitis, glucose, intra-amniotic infection

Infection may be a significant etiologic factor in the development of preterm labor (PTL) and preterm premature rupture of membranes (PPROM).[1] Amniocentesis is sometimes employed to obtain amniotic fluid for analysis from patients presenting with PTL or PPROM in order to exclude infection as an underlying cause.[1–4] Amniotic fluid culture has been utilized for the diagnosis of microbial invasion of the amniotic cavity; however, the results of bacteriologic cultures may take in excess of 2 days. In addition, biologic properties of amniotic fluid such as bacteriolytic/static activity

Address correspondence/reprint requests to Dr. Gary A. Dildy, Director, Perinatal Center, Utah Valley Regional Medical Center, 1034 North 500 West, Provo, UT 84604.

Received September 21, 1993
Accepted December 31, 1993

Clinical Study may potentially result in negative culture results.[5] The Gram stain is a rapid test that provides useful information for clinical management. However, at least $1 \times 10^5$ organisms/ml must be present to detect microbial invasion of the amniotic cavity by Gram stain, thus limiting the sensitivity of amniotic fluid Gram stain to 45%.[6]

Low glucose concentration has been used as a marker for detecting infection in cerebrospinal fluid (CSF).[7–10] Recently the use of amniotic fluid glucose concentration has been reported in the detection of microbial invasion of the amniotic cavity in patients with PTL and PPROM.[11–14] The purpose of this study was to compare the value of Gram stain of amniotic fluid and amniotic fluid glucose in the identification of microbial invasion of the amniotic cavity and the development of clinical chorioamnionitis in patients presenting with PTL or PPROM. The relationship between presenting clinical diagnoses (PTL, PPROM, PTL with PPROM) and incidence of positive diagnostic tests was also evaluated.

### MATERIALS AND METHODS

This study was conducted at Baylor College of Medicine's affiliated hospitals after approval by the Baylor College of Medicine and Harris County Hospital District institutional review boards. The period of research was between February and December of 1990. Patients with the primary diagnosis of PTL or PPROM who were undergoing amniocentesis to rule out microbial invasion of the amniotic cavity were asked to enroll in the study. Patients were enrolled if chorioamnionitis was suspected as an underlying cause of PTL or PPROM, but not if a clinical diagnosis of chorioamnionitis had already been made.

PTL was defined as regular painful uterine contractions with a frequency of at least three contractions within 10 minutes or documented cervical change. PPROM was confirmed by speculum examination with nitrazine and fern testing. The clinical diagnosis of chorioamnionitis was made according to the criteria described by Gibbs and coworkers[15] as a syndrome of fever, with either maternal or fetal tachycardia, uterine tenderness, foul-smelling amniotic fluid, or peripheral blood leukocytosis.

Patients with antecedent antimicrobial therapy within 1 month of presentation were excluded from the study. Patients were enrolled in the study regardless of previous tocolytic therapy, which included terbutaline or magnesium sulfate. Patients with known glucose intolerance were excluded from the study.

Amniocentesis was performed under local anesthesia and with ultrasound guidance. Samples of amniotic fluid were submitted to the hospital laboratory for clinically indicated tests such as Gram stain, culture and sensitivity, and pulmonary maturity studies.

The remainder of the amniotic fluid specimen was used for this study. Amniotic fluid was placed in a BBL Port-a-cul vial (Becton Dickinson and Company, Cockeysville, MD) and in separate sterile polypropylene tubes (Becton Dickinson Labware, Lincoln Park, NJ) that were immediately refrigerated and transported for storage in a −70°C freezer until glucose assay.

Gram stain was performed with reagents (BBL Microbiology Systems, Cockeysville, MD) under standard conditions. Quantitation of both microorganisms and white blood cells (WBCs) were as follows: 0, none present; < 1+, 1 to occasional/oil immersion field (OIF); 1+, 1/OIF; 2+, 2–5/OIF; 3+, 5–10/OIF; and 4+, > 10/OIF.

Culture media were obtained from Regional Media Laboratories, Inc. (REMEL, Lenexa, KS). Samples were placed on blood agar (Trypticase Soy Agar (TSA) with 5% sheep blood), chocolate agar, Thayer Martin agar (improved), and phenylethyl alcohol (PEA) agar with 5% sheep blood at 35°C in a $CO_2$ incubator. Amniotic fluid was plated on MacConkey's agar in a 35°C non-$CO_2$ incubator. Anaerobic cultures were plated on anaerobic KV blood agar [Centers for Disease Control (CDC) formulation] and anaerobic PEA blood agar (CDC formulation) and grown in an anaerobic chamber at 35°C. A-7 medium was utilized for the isolation of *Mycoplasma* and *Ureaplasma* (Scott Lab) and incubated at 35°C in $CO_2$. Quantitative amniotic fluid cultures were performed with a 1 μl/ml loop. Amniotic fluid cultures were considered positive if *Mycoplasma* or *Ureaplasma* were isolated.

Amniotic fluid glucose concentrations were determined in singlicate using the glucose oxidase method. Amniotic fluid glucose measurements were not used in the clinical management of patients. Subjects with positive amniotic fluid Gram stains were placed on antibiotics and then were delivered.

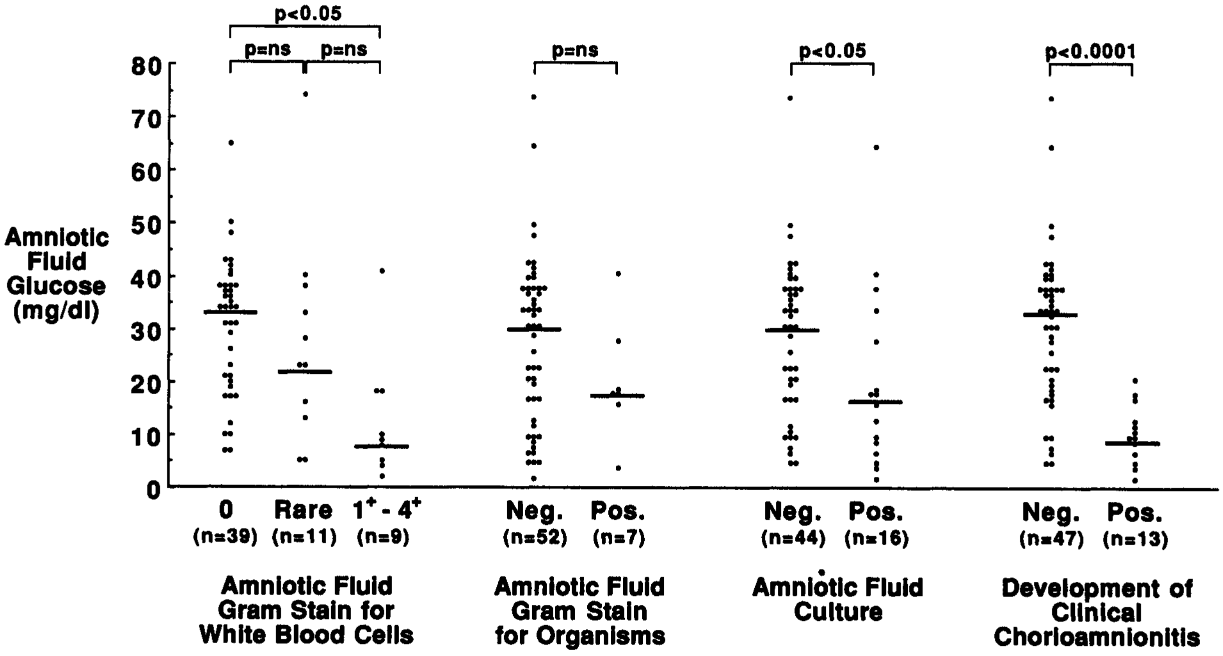

Fig. 1. Relationship of amniotic fluid glucose concentrations in patients with PTL and/or PPROM to amniotic fluid Gram stain, amniotic fluid culture, and development of clinical chorioamnionitis. Bars represent median values. $1^{+}$–$4^{+}$, see text for explanation.

Statistical analysis was performed using the Mann-Whitney test to compare non-parametric variables between the infected and non-infected groups. Fisher exact analysis (two-tailed test) was used to compare clinical outcomes between the groups with normal and low amniotic fluid glucose concentration. Diagnostic indices (sensitivity, specificity, positive predictive value, and negative predictive value) were used in the prediction of positive amniotic fluid cultures and development of clinical chorioamnionitis.

### RESULTS

Sixty women were enrolled in the study, 34 with PTL, 19 with PROM, and 7 with both PTL and PPROM. Median (range) maternal age was 25 years (15–41), and median (range) gravidity and parity were 2 (1–8) and 1 (0–6), respectively.

Subjects with positive amniotic fluid cultures had significantly lower ($P < 0.05$, Mann-Whitney) median amniotic fluid glucose concentrations (16.0 vs. 30.0 mg/dl), estimated gestational age at delivery (30.6 vs. 33.4 weeks), amniocentesis-to-delivery interval (9.8 vs. 174.0 hours), and birthweight (1,514 vs. 2,020 g). There was no statistical difference in serum WBC (12.9 vs. 11.6 $\times$ $10^3/mm^3$) or estimated gestational age at presentation (30.4 vs. 32.0 weeks).

Subjects who developed clinical chorioamnionitis had significantly lower median amniotic fluid glucose concentrations (9.0 vs. 33.0 mg/dl), estimated gestational age at presentation (29.7 vs. 32.0 weeks), estimated age at delivery (29.7 vs. 33.7 weeks), amniocentesis-to-delivery interval (8.5 vs. 203.0 hours), and birthweight (1,280 vs. 2,145 grams). There was no significant difference in serum WBC (14.8 vs. 11.3 $\times$ $10^3/mm^3$) at presentation.

Figure 1 depicts the relationship of amniotic fluid glucose concentration to amniotic fluid Gram stain for WBCs, amniotic fluid Gram stain for microorganisms, amniotic fluid culture, and the development of clinical chorioamnionitis. A significant difference ($P < 0.05$) was seen between patients with negative Gram stain for WBCs versus those with 1+ to 4+ WBCs. The median amniotic fluid glucose concentration for those patients with positive Gram stain for organisms was 17 mg/dl, while that of those with a negative Gram stain for organisms was 30 mg/dl.

**TABLE 1. Laboratory tests to predict positive amniotic fluid cultures[a]**

| | + Amniotic fluid culture | | | |
|---|---|---|---|---|
| | Sensitivity | Specificity | PPV | NPV |
| Amniotic fluid Gram stain for organisms | 43.8 | 100 | 100 | 82.7 |
| Amniotic fluid Gram stain for WBCs | 68.8 | 79.1 | 55.0 | 87.2 |
| Amniotic fluid glucose concentration < 15 mg/dl | 43.8 | 79.5 | 43.8 | 79.5 |

[a]PPV, positive predictive value; NPV, negative predictive value.

**TABLE 2. Laboratory tests to predict clinical chorioamnionitis[a]**

| | + Clinical chorioamnionitis | | | |
|---|---|---|---|---|
| | Sensitivity | Specificity | PPV | NPV |
| Amniotic fluid Gram stain for organisms | 21.4 | 95.2 | 60.0 | 78.4 |
| Amniotic fluid Gram stain for WBCs | 64.3 | 81.0 | 52.9 | 87.2 |
| Amniotic fluid culture | 60.0 | 88.1 | 64.3 | 86.0 |
| Amniotic fluid glucose concentration < 15 mg/dl | 73.3 | 88.1 | 68.8 | 90.2 |

[a]PPV, positive predictive value; NPV, negative predictive value.

The sensitivity, specificity, and positive predictive value (PPV) of amniotic fluid glucose to detect positive amniotic fluid culture were 43.8, 79.6, and 43.8%, respectively, at < 15 mg/dl; and 50.0, 79.6, and 47.1%, respectively, at < 16 mg/dl. The sensitivity, specificity, and PPV of an amniotic fluid glucose < 15 mg/dl to detect clinical chorioamnionitis were 73.3, 88.1, and 68.8%, respectively. Raising the cutoff point to < 16 mg/dl changed the sensitivity, specificity, and PPV to 76.9, 85.1, and 58.8%, respectively. A cutoff value for low amniotic fluid glucose concentration was chosen as < 15 mg/dl for comparison with other laboratory techniques. When cultures positive only for *Mycoplasma* and *Ureaplasma* were considered negative, the sensitivity and specificity of low amniotic fluid glucose at a cutoff of 15 mg/dl to detect positive cultures were 37.5% and 75%, respectively. This was not appreciably different from values obtained by considering the isolation of *Mycoplasma* and *Ureaplasma* as positive amniotic fluid cultures.

Validity analysis of amniotic fluid glucose and other laboratory tests for the prediction of positive amniotic fluid cultures and clinical chorioamnionitis are depicted in Tables 1 and 2. Amniotic fluid glucose appears to be more sensitive in detecting clinical chorioamnionitis than Gram stain for microorganisms and Gram stain for WBCs and amniotic fluid culture (Table 2). Low amniotic fluid glucose concentration appears to be a better predictor of the subsequent development of clinical chorioamnionitis (Table 2, sensitivity = 73.3%) than the development of positive amniotic fluid culture (Table 1, sensitivity = 43.8%).

Of the 60 study subjects, 44 had normal amniotic fluid glucose concentration and 16 had low amniotic fluid glucose concentration. There was a significantly increased incidence of clinical chorioamnionitis (68.8 vs. 9.1%, $P < 0.001$, Fisher exact test) and delivery within 72 hours (75.0 vs. 38.6%, $P < 0.004$) in the group with low amniotic fluid glucose concentration. The increased incidence of positive amniotic fluid cultures (43.8 vs. 20.5%) in the low amniotic fluid glucose group did not reach statistical significance.

Among the 16 patients who were found to have positive amniotic fluid cultures, 8 cultures were positive only for *Mycoplasma* spp. (Table 3). Thus, at least one-half of patients with positive cultures would be expected to have negative Gram stains for organisms. There were nine false negative and no false positive Gram stains for microorganisms. Nine of the 16 patients with positive cultures did not develop clinical chorioamnionitis. Of these nine patients, eight demonstrated normal amniotic fluid glucose concentration. There was no statistical difference between amniotic fluid glucose concentra-

TABLE 3. Results of 16 positive amniotic fluid cultures among 60 women with PTL and/or PPROM undergoing amniocentesis to rule out intra-amniotic infection[a]

| Pt | At amniocentesis | | Amniotic fluid Gram stain | | | AF glucose | |
|---|---|---|---|---|---|---|---|
| no. | Diagnosis | EGA (wks) | Organisms | WBC | Amniotic fluid culture | mg/dl | Clinical chorio. |
| 3 | PTL | 29.1 | NOS | 2+ | *Ureaplasma* | 4 | No |
| 5 | PTL | 32.4 | NOS | 0+ | *Ureaplasma* | 64 | No |
| 7 | PROM | 31.1 | NOS | 0+ | *Ureaplasma* | 33 | No |
| 10 | PROM | 33.9 | NOS | 0+ | *Ureaplasma* | 37 | No |
| 11 | PROM | 31.7 | NOS | 0+ | *Ureaplasma, N. gonorrhoeae* | 6 | Yes |
| 12 | PTL | 17.6 | NOS | 2+ | *Ureaplasma* | 9 | Yes |
| 14 | PROM | 30.3 | < 1+ yeast | 0+ | > 1 × $10^5$ *C. albicans* | 18 | No |
| 21 | PTL | 30.4 | NOS | 4+ | 1.8 × $10^4$ *C. ochracea* | 8 | Yes |
| 23 | PROM, PTL | 23.4 | 1+ GPC, 2+ GNR | 4+ | > 1 × $10^5$ *G. vaginalis* | 3 | Yes |
| 37 | PROM | 30.6 | NOS | < 1+ | *Ureaplasma* | 12 | Yes |
| 38 | PROM | 29.0 | 4+ GNDC | 3+ | > 1 × $10^5$ *N. gonorrhoeae* | 17 | Yes |
| 41 | PROM | 33.3 | 1+ GNR | < 1+ | 7.5 × $10^4$ *H. influenzae* | 15 | No |
| 45 | PTL, PROM | 26.3 | 4+ GPC | 1+ | > 1 × $10^5$ *S. agalactiae* | 17 | No |
| 55 | PROM, PTL | 29.7 | NOS | 4+ | *Ureaplasma* | 1 | Yes |
| 56 | PTL | 29.4 | 3+ yeast, 4+ GPR | 4+ | *Ureaplasma, Mycoplasma* | 40 | No |
| 58 | PTL | 31.1 | < 1+ GPR | | > 1 × $10^5$ *Lactobacillus* spp., | 27 | No |
| | | | < 1+ yeast | 1+ | 3 × $10^3$ *C. albicans* | | |

[a]NOS, no organisms seen; GPC, Gram positive cocci; GNR, Gram negative rods; GNDC, Gram negative diplococci; GPR, Gram positive rods; EGA, estimated gestational age; AF, amniotic fluid. For organism and WBC counts, see text.

tions in amniotic fluid cultures positive for *Mycoplasma* spp. versus bacterial species. No anaerobic organisms were isolated from culture.

Among the 16 patients with positive amniotic fluid cultures, 7 had PPROM, 6 had PTL, and 3 had PPROM with PTL. There was no significant difference in amniotic fluid glucose concentration between patients with PPROM and PTL (19.7 vs. 25.3 mg/dl; $P = 0.58$).

Amniotic fluid WBC count was lower in association with PPROM than with PTL; however, this did not reach statistical significance (0.57 + vs. 2.08 + ; $P = 0.077$). The relationships between primary clinical diagnoses (PTL, PPROM, or PTL with PPROM) and clinical tests or outcomes are shown in Figure 2.

Among the 16 patients with low amniotic fluid glucose (<15 mg/dl), 6 had negative cultures and did not develop chorioamnionitis. One patient with Rh alloimmunization prematurely delivered 18 hours following amniocentesis. One patient delivered in 6 hours with abruptio placentae, which was believed to be the underlying cause of PTL. Another patient with PTL delivered in 91 hours with fetal distress and suspected pregnancy-induced hypertension. A patient with PPROM and oligohydramnios delivered after 232 hours with fetal distress; the neonate was found to have congenital syphilis. Two patients with low amniotic fluid glucose and negative cultures had prolonged amniocentesis-to-delivery times of 36 and 44 days.

### DISCUSSION

The effects of leukocytes and bacteria on the glucose concentration of CSF in bacterial meningitis have been well established.[7] Both activated leukocytes and bacteria are required to lower CSF glucose concentration.[8–10] Altered glucose transport across inflamed membranes has also been a proposed mechanism for hypoglycorrhachia in bacterial meningitis.[16] This understanding makes it plausible that the presence of bacteria andactivated leukocytes in amniotic fluid may result in decreased concentrations of glucose.

Weiss and colleagues[17] studied 2,295 samples of amniotic fluid obtained between the 14th and 42nd weeks of pregnancy. Mean amniotic fluid glucose concentration in normal pregnancy rose between the 14th and 17th weeks of pregnancy and then declined steadily toward term. They reported the tenth percentile of amniotic fluid glucose concentration for normal pregnancies at 28/29, 30/31, 32/33, 34/35, 36/37, and 38/39 weeks of gestation to be 20, 18, 17, 16, 14, and 12 mg/dl, respectively. Amniotic fluid glucose concentration may be elevated in diabetic pregnancies and reduced in

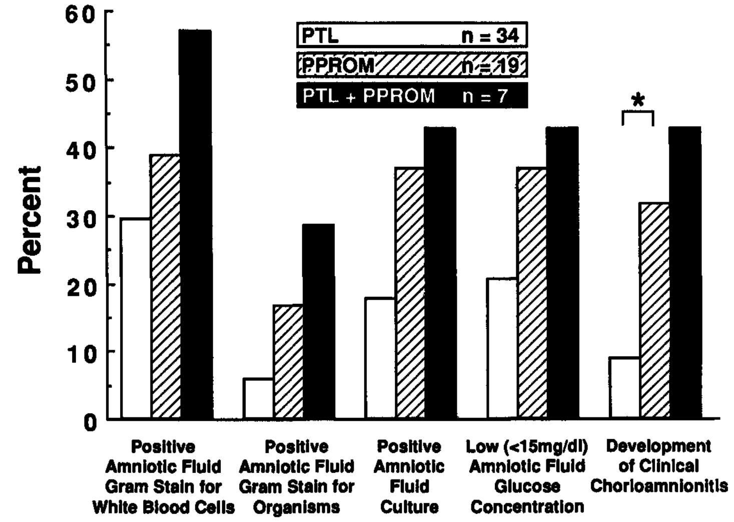

Fig. 2. Relationship of clinical diagnosis (PTL, PPROM, or PTL with PPROM) and incidence of positive clinical tests and development of clinical chorioamnionitis. The asterisk denotes $P < 0.05$, Fisher's exact test.

pregnancies complicated by growth retardation or fetal malformation.[17–19]

All papers published to date regarding amniotic fluid glucose concentration in detecting microbial invasion of the amniotic cavity have reported different thresholds of low amniotic fluid glucose.[11–14] Romero and colleagues[11] studied 168 women with PTL and intact membranes. They found that a cutoff of <14 mg/dl resulted in a reasonable compromise between the true positive and false positive rate. With this threshold, the sensitivity, specificity, and PPV were 86.9%, 91.7%, and 62.5%, respectively. Kirshon and associates[12] studied 39 patients with PTL and/or PPROM. All nine patients with microbial invasion of the amniotic cavity were noted to have amniotic fluid glucose concentrations of <10 mg/dl, and none with >10 mg/dl developed chorioamnionitis. Kiltz and coworkers[13] evaluated the amniotic fluid glucose concentrations of 84 patients who were either in PTL, near term but not in labor, or at term in labor. They found that an amniotic fluid glucose concentration ≤ 5 mg/dl had a PPV of 90% for positive culture and at > 20 mg/dl had a NPV of 98%. The PPV at ≤ 15 mg/dl was 46%, and the NPV at > 15 mg/dl was 97%. The PPV for amniotic fluid glucose concentration in our study was 43.8% at < 15 mg/dl and 47.1% at < 16 mg/dl, similar to the findings of Kiltz and co-workers.

In the clinical setting of PTL or PPROM, the ramifications of a false positive test include induction of labor and premature delivery, which are highly undesirable. Likewise, the results of clinical action based upon a false negative test may include unwarranted tocolysis, delayed delivery and withholding of antibiotic therapy, which may lead to adverse maternal and neonatal outcomes. We chose to use 15 mg/dl as a cutoff for normal amniotic fluid glucose concentration based upon the ability to predict positive cultures and the development of clinical chorioamnionitis. Analysis of our data demonstrates that this threshold for amniotic fluid glucose concentration is comparable or superior to the other laboratory tests evaluated for detection of clinical chorioamnionitis, but not for positive cultures. It should be recognized that immediate delivery for positive amniotic fluid Gram stain or culture may have a significant effect in reducing the number of subjects who would later develop clinical chorioamnionitis.

It appears that a cutoff for amniotic fluid glucose concentration at 15 mg/dl would be appropriate for general clinical use at this time. The use of a lower cutoff point would reduce the false positive rate at the expense of sensitivity, especially at earlier gestational ages. Studies examining the factor of gestational age have not yet been conducted. Gestational age may play an important role in interpretation of these data, since an amniotic fluid glucose concentration of 14 mg/dl may actually be normal at 35 weeks but considered low at 32 weeks of gestation. This is demonstrated by our data in Table 3: four patients (numbers 14, 38, 41, and 45) were found to have normal (≥ 15 mg/dl) amniotic fluid glucose concentrations in the presence of both positive Gram stain for microorganisms and positive amniotic fluid cultures. If gestational age were taken into consideration, these four patients would have been considered to have low amniotic fluid glucose. Further studies examining the effects of gestational age appear warranted. Due to limitations in sensitivity and specificity, we recommend that amniotic fluid glucose concentration should not be used alone in making clinical decisions, but should be factored in with clinical signs, symptoms, and other laboratory tests.

### REFERENCES

1. Garite TJ, Freeman RK: Chorioamnionitis in the preterm gestation. Obstet Gynecol 59:539–545, 1982.
2. Miller JM, Hill GB, Welt SI, Pupkin MJ: Bacterial colonization of amniotic fluid in the presence of ruptured membranes. Am J Obstet Gynecol 137:451–458, 1980.
3. Bobitt JR, Hayslip CC, Damato JD: Amniotic fluid infection as determined by transabdominal amniocentesis in patients with intact membranes in premature labor. Am J Obstet Gynecol 140:947–952, 1981.
4. Zlatnik FJ, Cruikshank DP, Petzold CR, Galask RP: Amniocentesis in the identification of inapparent infection in preterm patients with premature rupture of the membranes. J Reprod Med 9:656–660, 1984.
5. Bratlid D, Lindback T: Bacteriolytic activity of amniotic fluid. Obstet Gynecol 51:63–66, 1978.
6. Romero R, Emamian M, Quintero R, et al.: The value and limitations of the Gram stain examination in the diagnosis of intraamniotic infection. Am J Obstet Gynecol 159:114–119, 1988.
7. Goldring S, Harford CG: Effect of leukocytes and bacteria on glucose content of the cerebrospinal fluid in meningitis. Proc Soc Exp Biol Med 75:669–672, 1950.
8. Petersdorf RG, Garcia M, Swarner DR: Mechanism of hypoglycorrhachia in experimental pneumococcal meningitis. Proc Soc Exp Biol Med 102:669–672, 1959.
9. Petersdorf RG, Swarner DR, Garcia M: Synergistic action of pneumococci and leukocytes in lowering cerebrospinal fluid glucose. Proc Soc Exp Biol Med 103: 380–382, 1960.
10. Bretz G, Mauer AM: Glucose consumption by polymorphonuclear leukocytes in the cerebrospinal fluid of patients with bacterial meningitis. J Pediatr 70:767–771, 1967.
11. Romero R, Jimenez C, Lohda AK, et al.: Amniotic fluid glucose concentration: A rapid and simple method for the detection of intraamniotic infection in preterm labor. Am J Obstet Gynecol 163:968–974, 1990.
12. Kirshon B, Rosenfeld B, Mari G, Belfort M: Amniotic fluid glucose and intraamniotic infection. Am J Obstet Gynecol 164:818–820, 1991.
13. Kiltz RJ, Burke MS, Porreco RP: Amniotic fluid glucose concentration as a marker for intra-amniotic infection. Obstet Gynecol 78:619–622, 1991.
14. Gauthier DW, Meyer WJ, Bieniarz A: Correlation of amniotic fluid glucose concentration and intraamniotic infection in patients with preterm labor or premature rupture of membranes. Am J Obstet Gynecol 165:1105–1110, 1991.
15. Gibbs RS, Castillo MS, Rodgers PJ: Management of acute chorioamnionitis. Am J Obstet Gynecol 136:709–713, 1980.
16. Williams RDB: Alterations in the glucose transport mechanism in patients with complications of bacterial meningitis. Pediatrics 34:491–502, 1964.
17 Weiss PAM, Hofmann H, Winter R, Purstner P, Lichtenegger W: Amniotic fluid glucose values in normal and abnormal pregnancies. Obstet Gynecol 65:333–339, 1985.
18. Spellacy WN, Buhi WC, Bradley B, Holsinger KK: Maternal, fetal and amniotic fluid levels of glucose, insulin and growth hormone. Obstet Gynecol 41:323–331, 1973.
19. Drazancic A, Kuvacic I: Amniotic fluid glucose concentration. Am J Obstet Gynecol 120:40–48, 1974.
